# Mammalian predators and vegetated nesting habitat drive reduced protected area nesting success of Kentish plovers, Yellow Sea region, China

**DOI:** 10.1002/ece3.9884

**Published:** 2023-03-12

**Authors:** Donglai Li, Yu Bai, Weipan Lei, Pinjia Que, Yang Liu, Emilio Pagani‐Núñez, Huw Lloyd, Zhengwang Zhang

**Affiliations:** ^1^ Provincial Key Laboratory of Animal Resource and Epidemic Disease Prevention, College of Life Sciences Liaoning University Shenyang China; ^2^ Ministry of Education Key Laboratory for Biodiversity Science and Ecological Engineering, College of Life Sciences Beijing Normal University Beijing China; ^3^ Chengdu Research Base of Giant Panda Breeding Chengdu China; ^4^ Sichuan Key Laboratory of Conservation Biology for Endangered Wildlife Chengdu China; ^5^ Sichuan Academy of Giant Panda Chengdu China; ^6^ State Key Laboratory of Biocontrol, School of Ecology Sun Yat‐sen University Guangzhou China; ^7^ Department of Health and Environmental Sciences Xi'an Jiaotong‐Liverpool University Suzhou China; ^8^ School of Applied Sciences Edinburgh Napier University Edinburgh UK; ^9^ Centre for Conservation and Restoration Science Edinburgh Napier University Edinburgh UK; ^10^ Department of Natural Sciences, Ecology and Environment Research Centre Manchester Metropolitan University Manchester UK

**Keywords:** daily survival rate, Kentish plover, nature reserve, nest predation, nesting habitat, *Suaeda salsa*

## Abstract

Protected areas provide essential habitats for wildlife by conserving natural and semi‐natural habitats and reducing human disturbance. However, whether breeding birds vulnerable to nest predation can benefit from strict land management in the protected area is unclear. Here, we compare the nesting performance of two groups of a ground‐nesting shorebird, the Kentish plover (*Charadrius alexandrinus*), in the protected area (Liaohekou Natural Reserve, hereinafter PA), and the control non‐protected area (non‐PA) around the Liaohekou Natural Reserve, in the north of the Yellow Sea, China, and identify which environmental factors, such as nesting habitat and nest materials, influence the daily nest survival rate (DSR). We found similar nesting habitats in both study areas, dominated by bare land or *Suaeda salsa* grassland. However, DSR was lower in PA (0.91 ± 0.01) than in non‐PA (0.97 ± 0.01). Kentish plovers nesting in areas with vegetation cover experienced lower DSR than in bare lands in both areas, and nests built with materials of *S. salsa* sticks had the lowest DSR in the bare land. Data from infrared cameras confirmed relatively higher predator abundances and nest predation rates by nocturnal mammals, such as Eurasian badgers (*Meles meles*), in PA than in non‐PA, and this pattern was especially evident for plover nests located in *S. salsa* grassland. Our results suggest that Liaohekou Natural Reserve protected area may not necessarily provide safe nesting sites for Kentish plovers due to the abundance of generalist mammal nest predators. However, the PA includes about 80% of the nests from both locations. This means the contribution of the total number of successful nests continues to be much higher within PA, with the benefit for the species that this brings in terms of conservation. The variation and mechanisms underlying differences in the nest predator communities of PA and non‐PA deserve further study.

## Graphical abstract

The daily nest survival rate of Kentish plover nesting within the protected areas was lower than that outside the protected areas. Kentish plovers nesting in areas with vegetation cover dominated by *Suaeda salsa* experienced lower DSR than in bare lands. Higher predator densities of nocturnal mammals in the protected areas increased the nest predation rate of Kentish plovers.

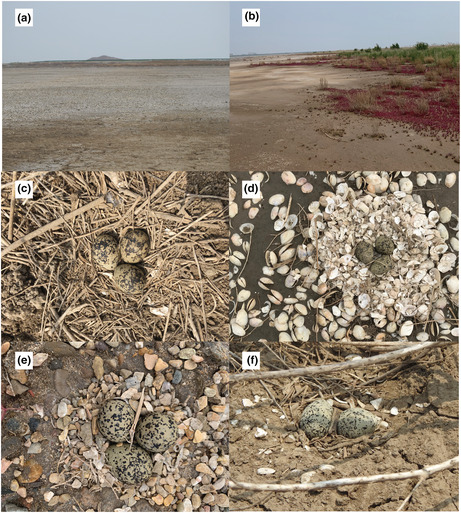

## 1 INTRODUCTION

Establishing protected areas is widely regarded as one of the most effective ways to safeguard distinct ecosystems and biodiversity (Gray et al., 2016; Kearney et al., 2020; Naughton‐Treves et al., 2005; Zheng et al., 2012). During the last two decades, the coverage of protected areas has grown rapidly worldwide (Cunningham & Beazley, 2018; de la Fuente et al., 2020; Watson et al., 2014). At present, protected areas, accounting for 18% of the land area in China, have contributed significantly to the conservation of wildlife and to enhance ecological diversity (Li & Pimm, 2020; MEP of PRC, 2020; Zhang et al., 2017). However, most of these protected areas targeted flagship or umbrella species (Wei et al., 2018). There are conflicting views on whether the protected areas function to conserve less charismatic species, especially when they are vulnerable to, e.g., predation or habitat change during the ecosystem restoration activities (Ainsworth et al., 2018; Li & Pimm, 2020; Rabearivony et al., 2010; Sergio et al., 2021; Wu et al., 2011).

For most wild birds, breeding performance is the most critical factor determining life‐history characteristics and population dynamics, which can be affected by a range of environmental factors at different spatial scales (Gómez et al., 2018; Wu et al., 2020). Generally, avian nesting success can be substantially influenced by nest site habitat selection, which is tightly linked to vegetation characteristics (Chotprasertkoon et al., 2017). Many ground‐nesting birds minimize predation risk through a range of adaptations related to vegetation use (Bures & Pavel, 2003; Martin, 1993; Massaro et al., 2013; Solis & de Lope, 1995). For example, some species of shorebirds conceal their nests in dense vegetation, and this greater nest concealment affords protection against predators (crypsis strategy: Engel et al., 2020). However, this same vegetation may also prevent birds from detecting approaching predators (predator detection strategy: Anteau et al., 2012; Gómez‐Serrano & López‐López, 2014; Lomas et al., 2014). This issue may be relevant depending on the predator community and the risk of predation at each stage of reproduction (Martin, 1988). Furthermore, the selection of vegetated nesting habitat by most shorebirds also restricts the trade‐off between predation pressure and effective thermoregulation, particularly for populations breeding in the low‐medium latitudinal area, where they often encounter hot temperatures in summer (Lomas et al., 2014). In turn, nest materials also have a critical influence on breeding performance, since the selection of different nest materials by shorebirds is not only determined by the availability of materials (Suárez et al., 2010) but may also relate to antipredator defense if the materials (e.g., vegetated material and shells) can enhance egg camouflage (Borges & Marini, 2010; Lee et al., 2010; Skrade & Dinsmore, 2013).

Variability in the biotic environment (e.g., nest predation and nesting density) among populations is common, even at small regional scales (Beauchamp, 2015; Small et al., 2007). Patterns of predation pressure can be determined by regional variation in predator communities, with nocturnal mammals considered important nest predators for ground‐nest birds, particularly in the natural or semi‐natural habitats within the protected area (Ellis et al., 2018; Gómez‐Catasús et al., 2021; Pol et al., 2022). In comparison, nesting density is primarily related to local habitat characteristics, in particular playing a vital role for colonial breeding ground‐nesting birds, gaining social anti‐predated vigilance from other nests. Furthermore, increasing human disturbance and landscape heterogeneity have reshaped patterns of nest site selection and nest predator communities, resulting in habitat mosaics with regional differences in breeding densities and nest predation risks (Nahid et al., 2020; Williams et al., 2009). Therefore, the landscape composition of protected areas situated in regions with adjacent and differently managed non‐protected areas with equivalent habitat types provides an ideal model landscape with which to examine how ground‐nesting birds' breeding performance in taxa such as shorebirds – which tend to have low survival rates (e.g., Que et al., 2015) – is affected by the protected area versus non‐protected area management regimes.

The conservation value of coastal wetlands along the Yellow Sea of China as a stopover site for large amounts of migratory shorebirds on the East Asian–Australasian Flyway has long been recognized, and consequently, many protected areas have been established to conserve these populations (China Coastal Waterbird Census Group et al., 2015; Hu et al., 2020; Ma et al., 2019). However, the conservation importance of different wetland habitats both inside and outside of protected areas for shorebird breeding populations still needs to be emphasized (Ma et al., 2019). Large populations of shorebirds (e.g., Kentish plover *Charadrius alexandrinus*; Que et al., 2015) and gulls (e.g., Saunders's gull *Saundersilarus saundersi*; Jiang et al., 2010) breed in this region, with a proportion of these populations nesting outside of the protected area networks. These “unprotected” breeding populations outside protected areas might experience various risks, mostly linked to human‐induced impacts, such as egg harvesting (Que et al., 2015) and a high risk of exposure to domestic mammals (e.g., cats, Dowding & Murphy, 2001; Loyd et al., 2013).

The large coastal wetland area along the Yellow Sea in China is an important migratory stopover and breeding site for Kentish plovers where they tend to breed in open habitats and nest in sandy bare land partially covered by stones and mollusks shells, and sometimes in saltmarsh habitats with sparse vegetation (Lei, 2010). Kentish Plovers have a polygamous mating system, and nests are incubated either by a single or both parents (Székely, 2019). The mode clutch size of Kentish plovers breeding in the Yellow Sea is three, and incubation lasts 27 days (Que et al., 2015).

In this study, we compare differences in nesting performance between two groups of Kentish plovers – one within Liaohekou Natural Reserve (protected area: PA) and the second group breeding outside Liaohekou Natural Reserve (non‐protected area: non‐PA) by taking into account the potential effects of nesting habitat, nest materials, and local predator communities. *Suaeda salsa* grasslands are typical breeding habitats for many waterbird species along the Yellow Sea's coast (Huang, 2017; Tian, 2002). Previous observations of the Kentish plover population have revealed that this species also uses this habitat for nesting, even though they tend to use bare land in other regions (Amat & Masero, 2004; Gómez‐Serrano & López‐López, 2014; Lei, 2010).

For this study, we formulated four predictions. Firstly, we expected higher nest success (i.e., higher daily nest survival rate: DSR) in the PA population as a result of habitat protection and restoration; secondly, we expected the daily nest survival rate of Kentish plovers nesting in the *S. salsa* would be lower in *S. salsa* habitat than in areas of their more traditional and evolved adapting breeding habitat of bare ground (Gómez‐Serrano & López‐López, 2014); thirdly, DSR would be influenced by nest material selection in different habitats because of the distinct color contrast between *S. salsa* vegetation and bare ground; finally, we expected that the abundance of natural nest predators in the PA would be significantly higher than in the non‐PA because of reduced human disturbance.

## 2 METHODS

### 2.1 Study area and species

**FIGURE 1:**
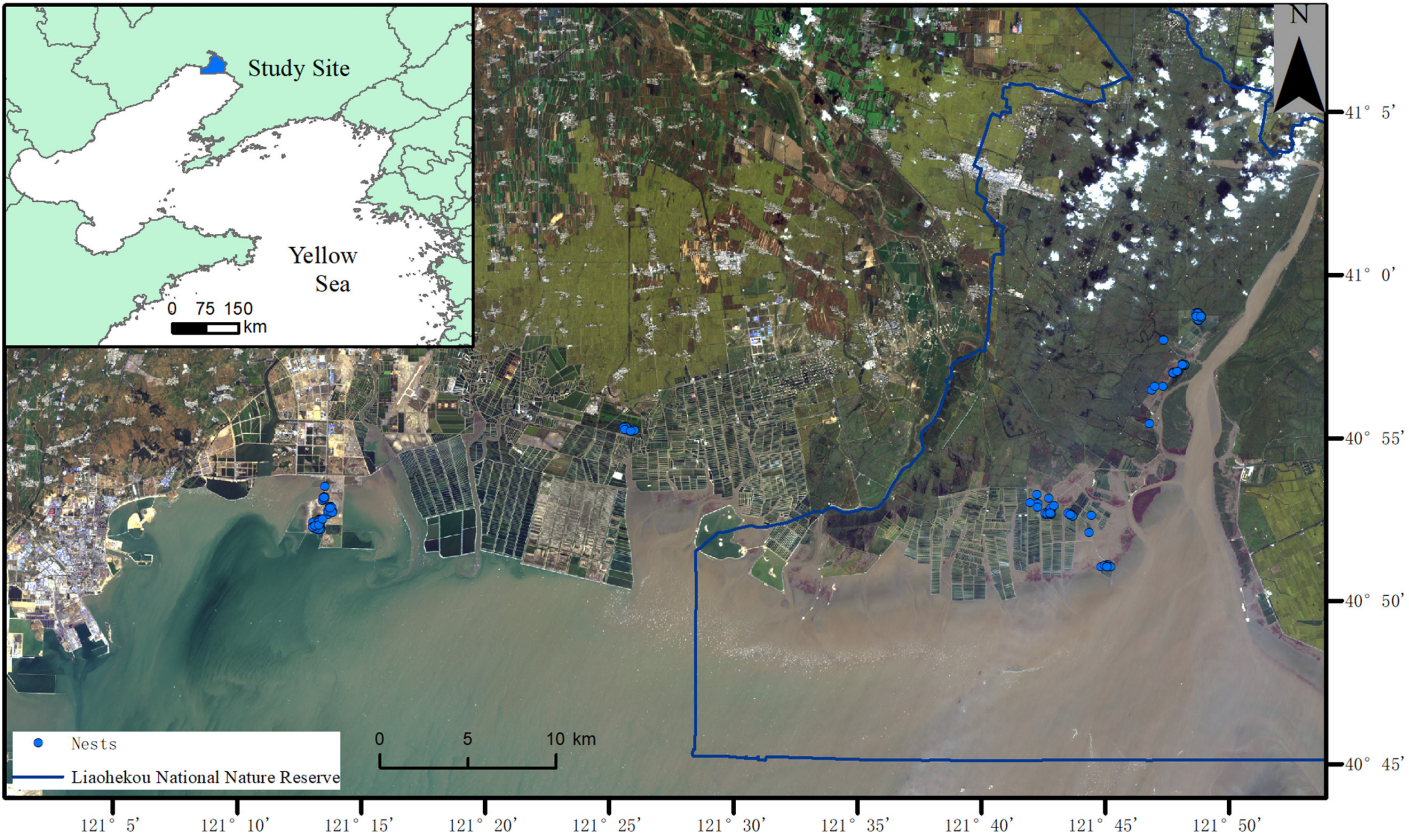
Nest site distribution of Kentish plovers breeding in the protected areas (PA) at Liaohekou Natural Reserve and the non‐protected areas (non‐PA) around the nature reserve, Liaoning, China.

**FIGURE 2:**
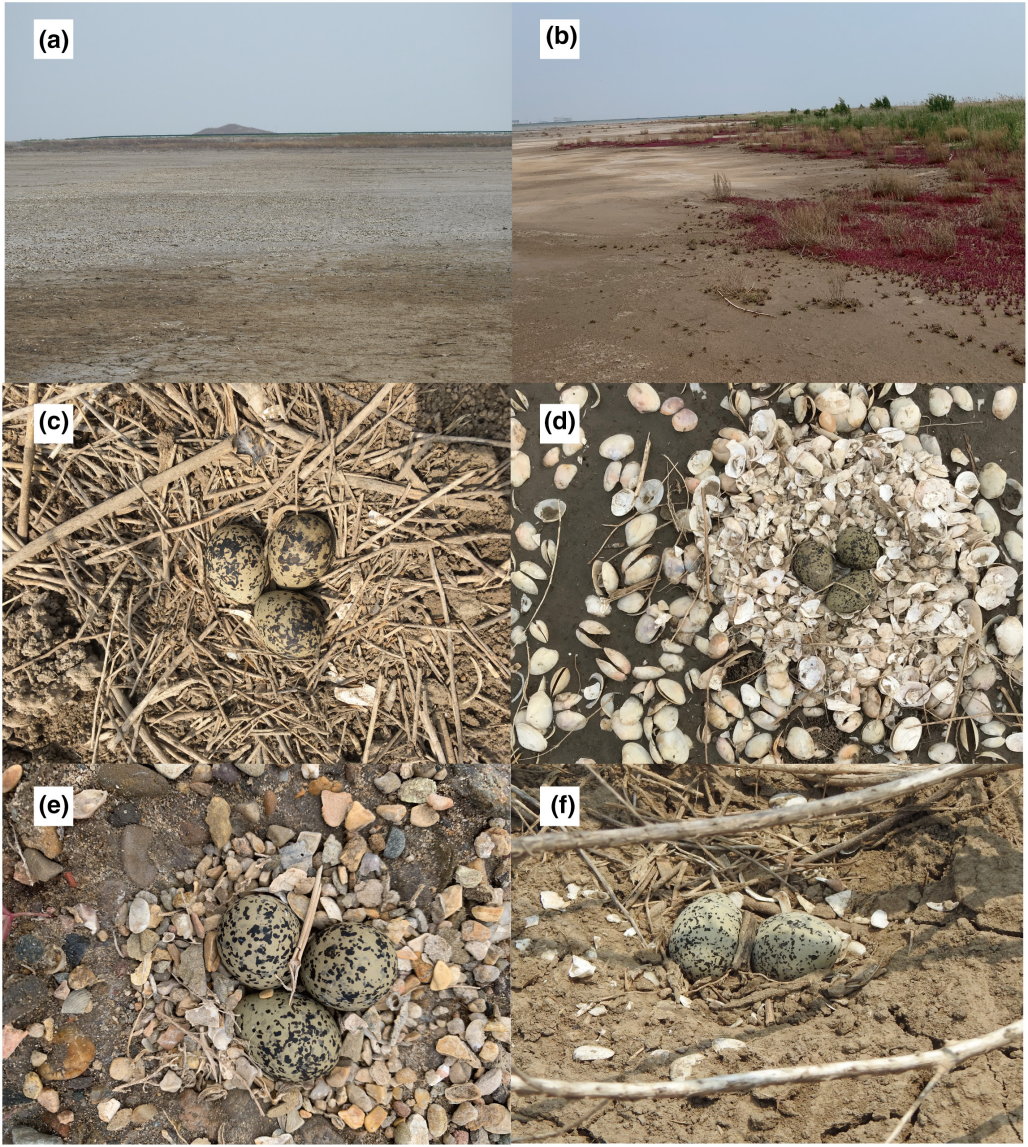
The nest habitats (a: bare land; b: vegetation) and nest materials (c: plant materials, *n* = 53; d: mollusks shells, *n* = 122; e: stones, *n* = 52; f: others, *n* = 38) of Kentish plover, Liaoning, China.

This study was conducted in May–July 2018–2019 and 2021, at the protected areas in Liaohekou National Natural Reserve (hereafter PA: 121°50.5′E, 40°33.5′N) and the non‐protected areas (non‐PA) around the Natural Reserve during 2020–2021, Liaoning Province, China (Figure 1). Uneven sampling between the two areas was caused by the cessation of research licenses being issued for the nature reserve in 2020 due to the COVID‐19 pandemic, and no permissions were granted to access the private land of the non‐PA during 2018 and 2019. The breeding sites in the PA are located on the west side of the Liaohe river, mainly composed of bare land habitat with sparse *S. salsa* vegetation restored from formerly used/abandoned local fishery shellfish ponds since 2016 and 2017 (Zhang et al., 2021). Following the abandonment of the small‐scale shellfish ponds, these sites were restored primarily to recreate breeding areas for the endangered and globally threatened Saunders Gull, but have since also been colonized by several other breeding shorebird species including Kentish plover and Pied avocet (*Recurvirostra avosetta*). The breeding sites in non‐PA are composed of typical saltmarsh habitats with sparse vegetation, dominated by *S. salsa* and some abandoned fishponds. These areas were selected as a “control area” based on the extent and proportion of similar habitats found within the PA, that is, a landscape composed of sparse *S. salsa* vegetation and bare land (Figure 2a,b). The study areas covered approximately 130.0 and 70.7 ha situated in the PA and 59.3 ha located within the non‐PA. Average annual precipitation in the study area is 620–730 mm, and it rains mainly between June and September (http://www.nre.cn/html/04/bhq/2004‐11‐04‐14191.htm).

### 2.2 Nest monitoring and habitat assessment

All the selected breeding habitats of Kentish plover were systematically searched for nests between May and July each year. When nests with at least one egg were found, a Handheld GPS (Garmin 62) was used to record its location. Each nest was photographed using a digital camera (Nikon J5) to record the nesting environment and the composition of nest materials. Eggs were floated to estimate the incubation stage following the technique by Hays and Lecroy (1971). Nests were inspected one to two times per week during the early incubation stage (<22 days after egg‐laying) and at 1–2 days intervals after 22 days of incubation (26 days) (Que et al., 2015). We limited the time observers spent in proximity to each nest to no more than 5 min to minimize potential disturbances and the chances of nest abandonment. Nest fate was categorized as follows: (A) Failure: nests were considered to have failed when (1) eggs were observed being collected or destroyed by humans; (2) nests were considered predated when there was evidence of predation, for example, camera images, yolks, and egg content remaining in/around the nests; (3) were washed away by water or buried by mud due to flooding events and bad weather; and (4) were abandoned (i.e., nests in which eggs were still present but were cold for two nest‐checking periods). (B) Success: nest fate was considered to be successful when: (1) at least one nestling left the nest, (2) all eggs disappeared within 2 days of the estimated date of hatching, and did not meet any of the four criteria for “failure” as mentioned above. (C) Unknown: nest fate was considered unknown when: the above‐mentioned failure and success judgment criteria could not determine the fate of the nests. Nests with unknown fate (4.2%, *n* = 12) were not included in the subsequent statistical analyses (e.g., nest success rate).

Nesting habitats were recorded as either vegetated or bare land (Figure 2a,b). All the nests with at least 50% vegetation coverage within a 30 cm radius were classified as vegetated habitats. The vegetation covering the nests was mostly *S. salsa* (85.4%, *n* = 82), while short reed (*Phragmites australis*) represented the other 14.6% (*n* = 14). We estimated nest concealment by assessing visual obstruction by vegetation in five directions (up, N, E, S, and W) (Zero (0) = no vegetation cover in all directions, 5 = shielded in all directions) following Burhans and Thompson (2001). Most plover nests were dominated by one particular suite of substrates shells, stones, or plant materials (the latter of which mainly consisted of dead *S. salsa* stems). We categorized the nests from the digital photographs using these criteria. Nests classified as “others” were mainly composed of mud (Figure 2c–f). In addition, for each nest, we also recorded the closest distance to the nearest road, water edge, mudflat, coastline, and PA boundary, which were estimated from updated high‐resolution satellite images (http://www.sasclouds.com) using Arc GIS (v 10.2). Due to the fact that some plover nests were located outside the PA, we used the negative value to represent the relative distance to the PA boundary.

The distance matrix between each nest was calculated using R package “geosphere”(version 1.5‐14). The nearest neighborhood distance was defined as the shortest distance between conspecific nests during the active period. In addition, an annual aggregation index for each nest relative to the spatial distribution of all Kentish plover nests was calculated using the formula Σ exp (−*d*_*ij*_) (with *i* ≠ *j*), where *d*_*ij*_ was the linear distance between nests *i* and *j* (Hernández‐Brito et al., 2020).

### 2.3 Nest predators' monitoring

Sixty nests (21.0% of the total: *n* = 285) were randomly selected to be monitored using infrared cameras (Forsafe H801) in an effort to record nest predation events and identify the predator species during 2018–2021. Six nests failed as a result of a flooding event soon after the monitoring began. Infrared cameras were set about 1 m from the nest and fixed 20–30 cm above the ground on a wooden stick (Weston et al., 2017). The cameras were set to infrared trigger mode and programmed to capture at least two images and a video of 10 s. Cameras were visited every 5–10 days to check and replace batteries and SD cards. The nest predation, nest predator species, and predation time were identified from the video (or photos). Other potential nest predators did not damage the nest but were captured by the cameras to reflect relative predator abundances. A number of studies have reported no negative effects of infrared camera monitoring on the nest survival of shorebirds (e.g., Ellis et al., 2018; Mcguire et al., 2022; Salewski & Schmidt, 2022). In fact, in our study, the nest predation rate for the monitored nests (33.3%) was significantly lower than the non‐monitored nests (59.8%, *χ* ^2^ = 11.211, df = 1, *p* < .001) in our limited sampling. If the same predator species was photographed in the same aggregated nesting place >30 min from a previous recording, then we considered this as an independent photograph (IP). Camera day (CD) was defined as one camera working for 24 h. The photographic rate (PR) was used as the relative abundance of predators (Guo et al., 2016) and was calculated as (number of IP × 100)/CD.

### 2.4 Statistical analyses

Variability of the proportion of each variable (i.e., nesting habitat) between the PA and non‐PA was analyzed using Chi‐square tests. Potential effects of the protected area and other covariates (Table 1) on daily nest survival rate (DSR) were fitted with RMark v2.2.7 in R v4.0.2, which interfaces with Program MARK (Pierce et al., 2019; Weintraub et al., 2016; White & Burnham, 1999). We used two multicollinearity tests to calculate generalized variation inflation factors (GVIF) between all independent variables except for either protection status or distance to PA boundary was considered in R (R Core Development Team v4.0.2). Variables with a GVIF larger than 10 were eliminated from the models due to collinearity issues (Zhao et al., 2020). There was significant collinearity between two categorical factors, year and protected status. However, we found no significant annual variation in the nest success rate in both regions (see Section 3), which implied that the main source of nest survival difference originated from the protected status of the area rather than an annual effect. For these reasons, we decided to remove year (GVIF > 38) while other independent variables performed well in both multicollinearity tests (Table S1).

**TABLE 1:** Descriptions of protection effort and nesting habitat of Kentish plover in analyzing the DSR of Kentish plover.

Variables	Description
Year	2018–2021
Day	Day of the breeding season
Protection status (Prot)	The protection status of nest sites either within (PA) or outside the Liaohekou National Nature Reserve (non‐PA)
Habitat (Hab)	Nesting habitats of Kentish plovers either in the vegetated (dominated mainly by *S. salsa*) or bare land
Nest materials (NM)	The nest materials of Kentish Plovers are divided into four types (mollusks shells, stones, plant materials, and others, i.e., mud)
Nest concealment (Con)	Concealment of the nest between 0 and 5
Neighbors distance (Neighbor)	The shortest distance to the nearest active Kentish plover nests
Aggregation index (AI)	An annual aggregation index for each nest relative to the spatial distribution of all Kentish plover nests
Distance to road (road)	The shortest distance to the nearest road
Distance to water (water)	The shortest distance to the nearest water edge
Distance to mudflat (dis_mud)	The shortest distance to the edge of mudflat
Distance to coastline (dis_coast)	The shortest distance to the coastline
Distance to PA boundary (dis_PA)	The shortest distance to the boundary of the nature reserve. The values for the nests outside the nature reserve were minus

To identify potential factors influencing nest DSR, we built a set of candidate models with a single explanatory variable. We found that distance to the nearest water edge (Water), distance to the nearest road (Road), and the nearest neighborhood distance (Neighbor) received less support (sum of models weight < 0.0001). Thus, these factors were not included in the following combined models. Nest age should be an important factor in quantifying nest survival (e.g., Weiser, 2021) but we were unable to incorporate this into our models due to the loss of data for 30% of nests due to a technological error. All other variables (except for distance to PA boundary) were used to build a subset of models, including all possible combinations and the two‐way interactions between each of the four factors (protected status (Prot), day of the breeding season (Day), nest material (NM), and nesting habitat (Hab)) based on predictions. To account for model selection uncertainty, we model‐averaged parameter estimates from models within 2 AIC units of the best model in the final set in the MuMIn package (Bartoń, 2015) and report them as means ± standard error (SE), 95% confidence intervals (CI), and Wald test *z*‐scores (Bartoń, 2015). We re‐fit the models replacing the categorical variable of protection status with the continuous variable of distance to PA boundary, and yielded the same conclusion (Appendix S1).

## 3 RESULTS

### 3.1 Nesting habitat selection, breeding population size, and nest success in PA and non‐PA

**FIGURE 3:**
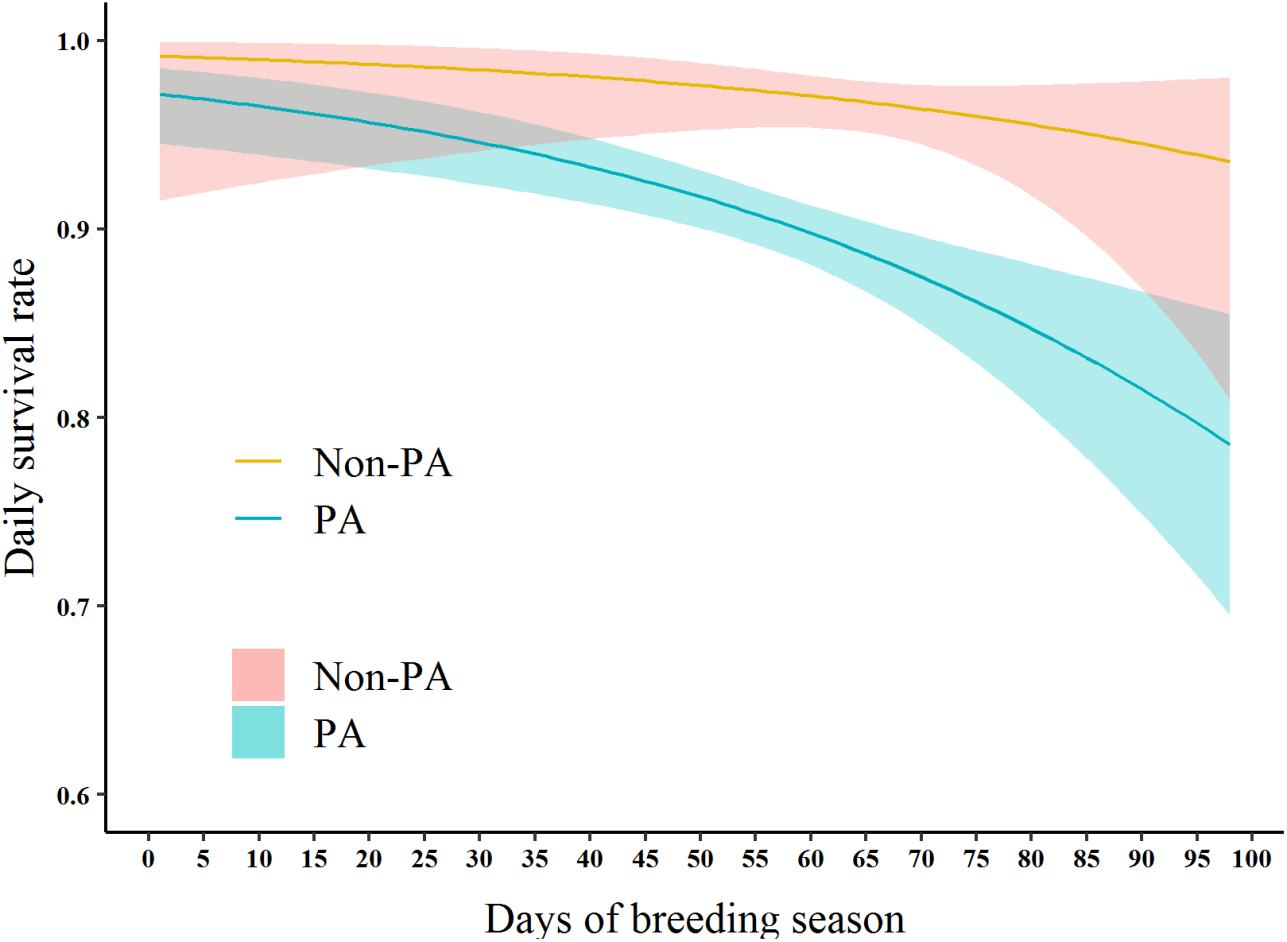
Relationship between daily survival rate and breeding season days with 95% confidence intervals in a protected area (PA) and non‐protected area (non‐PA) in Liaoning, China.

In total, 285 Kentish plover nests were found, with 225 (78.9%) nests in the PA and 60 (21.1%) nests in the non‐PA. The percentage of nests in the vegetated habitat (96, 33.7%) showed no significant differences between the two regions (non‐PA: 45.0% vs. PA: 33.7%; Chi‐square tests: *χ* ^2^ = 3.263 df = 1, *p* = .071; Table 2). The nest density of Kentish plovers found in the PA (1.2 nests/ha) was higher than that in the non‐PA (0.5 nests/ha) using the same nest‐searching method. The proportion of three types of nesting materials (plant materials, mollusk shells, and stones) and “other” was significantly different between PA and non‐PA nests (Figure 3; *χ* ^2^ = 45.03, df = 3, *p* < .001).

**TABLE 2:** Summary of Kentish plover nests fates monitored in vegetated and bare land habitats in a protected area (PA) at Liaohekou Natural Reserve and a non‐protected area (non‐PA) around Xiaoling River Estuary, Liaoning, China.

Nest fates		Number of nests in PA (%)		Number of nests in non‐PA (%)		Total
		Vegetated habitat	Bare land	Vegetated habitat	Bare land	
Success	Success	8 (11.6%)	43 (29.9%)	11 (40.7%)	22 (66.7%)	84 (30.8%)
Failure	Predation	39 (56.5%)	87 (60.4%)	14 (51.9%)	9 (27.3%)	149 (54.6%)
	Washed away	11 (15.9%)	9 (6.3%)	0	0	20 (7.3%)
	Collected or destructed by human	9 (13.0%)	1 (0.7%)	0	1 (3.0%)	11 (4.0%)
	Abandoned	2 (2.9%)	4 (2.8%)	2 (7.4%)	1 (3.0%)	9 (3.3%)
Total		69 (100%)	144 (100%)	27 (100%)	33 (100%)	273 (100%)

*Note*: The nest fate of 12 nests was unknown in the protected area, with 6 and 6 nests in both the vegetated habitat and bare land, respectively.

The apparent nest success rate of Kentish plovers was 30.8% (*n* = 273), being significantly higher in the non‐PA (55%) than the PA (23.9%, *χ* ^2^ = 21.196, df = 1, *p <* .0001). There were no significant annual variations in the apparent nest success rate in both the non‐PA (2020: 56.3%, 2021: 75%, *χ* ^2^ = 0.372, df = 1, *p* = .54) and the PA (2018: 15.9%, 2019: 30.5%, 2020: 34.8%, *χ* ^2^ = 5.658, df = 2, *p* = .06). Nest predation accounted for 78.8% (*n* = 189) of nest failures, and was significantly higher in the PA than that in the non‐PA (PA: 59.2%, non‐PA: 38.3%, *χ* ^2^ = 8.187, df = 1, *p* = .042). Nest failure due to flooding, human destruction, and abandonment is shown in Table 2.

### 3.2 Effects of protected status, nesting habitat, and nest material on the nest survival of Kentish plovers

**FIGURE 4:**
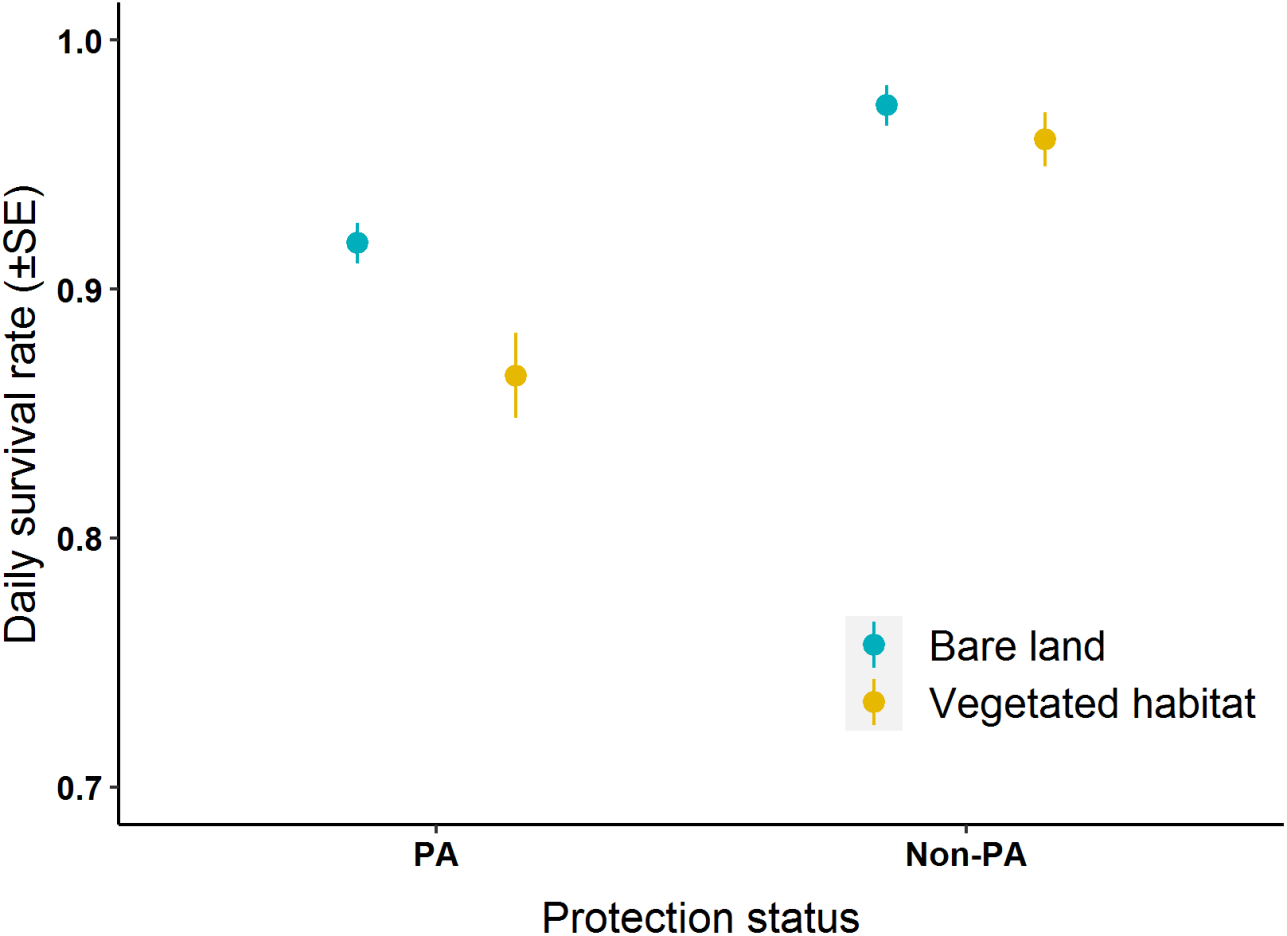
The daily survival rate of Kentish plovers in vegetation and bare land habitat in protected areas (PA, *n* = 205) and non‐protected areas (non‐PA, *n* = 60).

Three of our candidate models fitted the criterion of ΔAIC_c_ ≤ 2. The alternative models included protection status, day of the breeding season, nesting habitat, nesting material, aggregation index, and nest concealment (Table 3). There was a significant difference in the DSR of Kentish plovers between the PA and non‐PA, with the DSR in the PA (DSR: 0.91 ± 0.01) being significantly lower than in the non‐PA (0.97 ± 0.01; parameter estimated: *β* (SE): −1.28 (0.29); 95% CI = −1.87, −0.71; Table 4, Figure 4). There was also a significant negative effect of the distance to the PA boundary on the DSR of Kentish plovers (*β* (SE): −0.00004 (0.00001); 95% CI = −0.00006, −0.00002; *z* = 4.213, *p* < .001; Table S2, Figure S1). Day of the breeding season also had a significant adverse effect on the DSR of Kentish plovers (*β* (SE): −0.02 (0.01); 95% CI = −0.04, −0.01; Table 4), especially for the DSR in the PA (DSR: 0.97 ± 0.01 to 0.80 ± 0.04), which declined faster than that in the non‐PA (0.99 ± 0.02 to 0.96 ± 0.03; Figure 4).

**TABLE 3:** Three alternative models investigating the effects of breeding site, nesting habitat, and nest materials on the daily survival rate of the Kentish plover nests (*n* = 265) during 2018–2019, Liaoning, China. Models are ranked by differences in Akaike's information criterion (∆AIC_c_).

Model^a^	*K* ^b^	AIC_c_	Delta AIC_c_	Weight
S (~Day + Prot + Hab + NM + Hab*NM + AI)	11	693.36	0	0.47
S (~Day + Prot + Hab + NM + Hab*NM)	10	693.9	0.54	0.36
S (~Day + Prot + Hab + NM + Hab*NM + AI + Con)	12	695.27	1.91	0.18

^a^ Variable abbreviations: days of the breeding season (Day), protection status (Prot), nest materials (NM), habitat (Hab), and concealment (Conc).

^b^ *K* = Number of parameters.

**TABLE 4:** Beta estimates and standard errors with 95% confidence interval (CI) for covariates of daily survival rate of Kentish plover. For abbreviations of covariates can be found in Table 2. The referenced categories for the fixed factors of protection status, habitat, and nest material were “Non‐PA,” “bare land,” and “plant materials,” respectively.

Parameters	Estimate	SE	LCL	UCL	*z* Value	*p*
Intercept	**3.896**	**0.593**	**2.734**	**5.059**	**6.567**	**<.001**
Day	**−0.024**	**0.007**	**−0.037**	**−0.011**	**3.575**	**<.001**
Protection status: PA	**−1.282**	**0.291**	**−1.851**	**−0.712**	**4.409**	**<.001**
Hab: vegetation	−0.791	0.407	−1.590	−0.008	1.940	.042
NM: others	**1.413**	**0.417**	**0.595**	**2.230**	**3.388**	**.001**
NM: stones	**1.941**	**0.469**	**1.021**	**2.860**	**4.136**	**<.001**
NM: mollusks shells	**1.426**	**0.345**	**0.749**	**2.104**	**4.126**	**<.001**
AI	−0.128	0.084	−0.292	0.037	1.523	.128
Hab (vegetation): NM (others)	−1.151	0.603	−2.333	0.032	1.907	.057
Hab (vegetation): NM (stones)	**−1.839**	**0.598**	**−3.013**	**−0.665**	**3.071**	**.002**
Hab (vegetation): NM (mollusks shells)	**−1.829**	**0.491**	**−2.792**	**−0.865**	**3.720**	**<.001**
Con	0.266	0.451	−0.619	1.151	0.589	.556

Significances of *p* values < .005 was bold.

**FIGURE 5:**
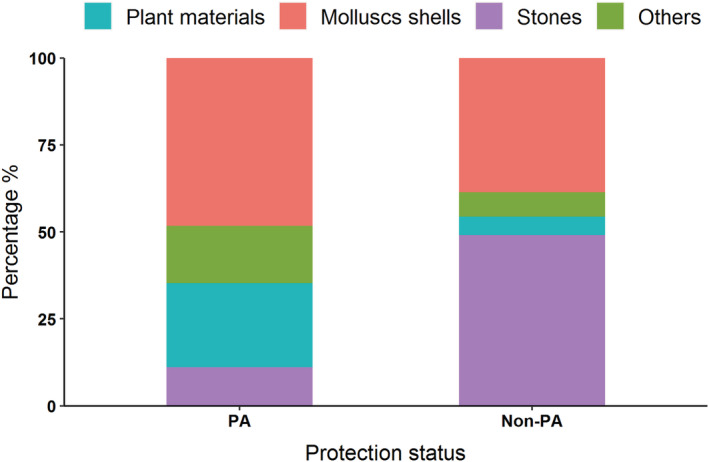
The proportions of nests with different materials (mollusks shells, stones, and plant materials) of Kentish plovers in a protected area (PA) and non‐protected area (non‐PA).

**FIGURE 6:**
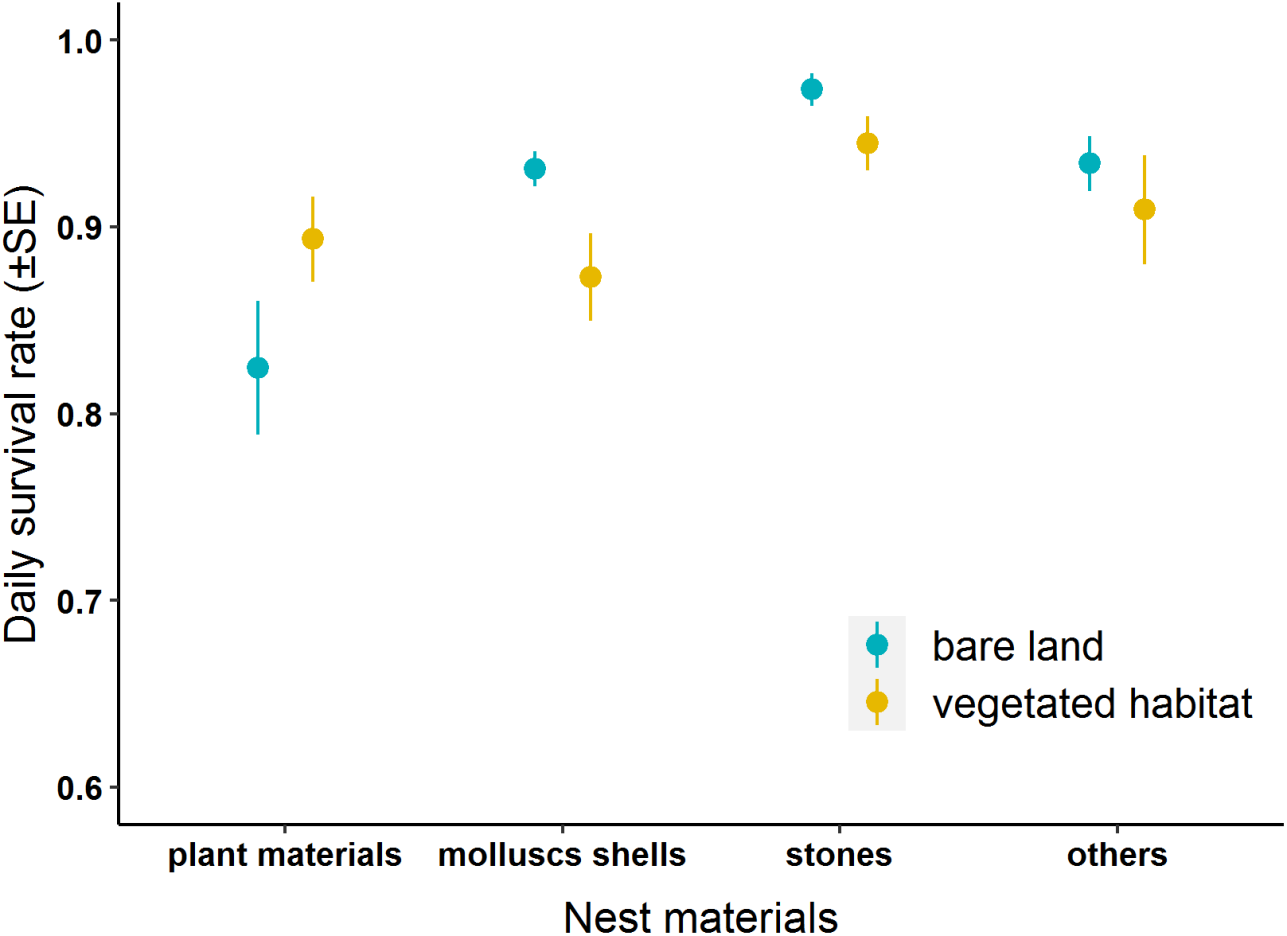
The daily survival rate of Kentish plovers in bare land (*n* = 173) and vegetation habitat (*n* = 92) with different materials (plant materials, mollusks shells, stones, and others).

DSR of Kentish plovers was also affected by nesting habitat, with DSR in the vegetated habitat (0.91 ± 0.01) being significantly lower than that in the bare land for the two study regions (0.94 ± 0.01; *β* (SE): −0.79 (0.41), 95% CI = −1.59, −0.01; Figure 5). There was no significant interaction between protection status and nesting habitat (*β* (SE): −0.49 (0.54), 95% CI = −1.56, 0.57). Furthermore, there were significant effects of nest material and the interaction between nesting habitat and nest material on the DSR of Kentish plovers (Figure 6). Nests built with plant materials experienced relatively lower DSR (0.86 ± 0.02), especially for the population nesting in the bare land (0.82 ± 0.04) compared with vegetated habitat (0.89 ± 0.02). On the contrary, nests with the other three types of materials experienced relatively higher DSR in the bare land than in vegetated habitats, except for “others” (Figure 6). No significant effects of nest concealment and aggregation index on the DSR of Kentish plovers were found (Table 4).

### 3.3 Nest predator composition and predation pressure between PA and non‐PA

**FIGURE 7:**
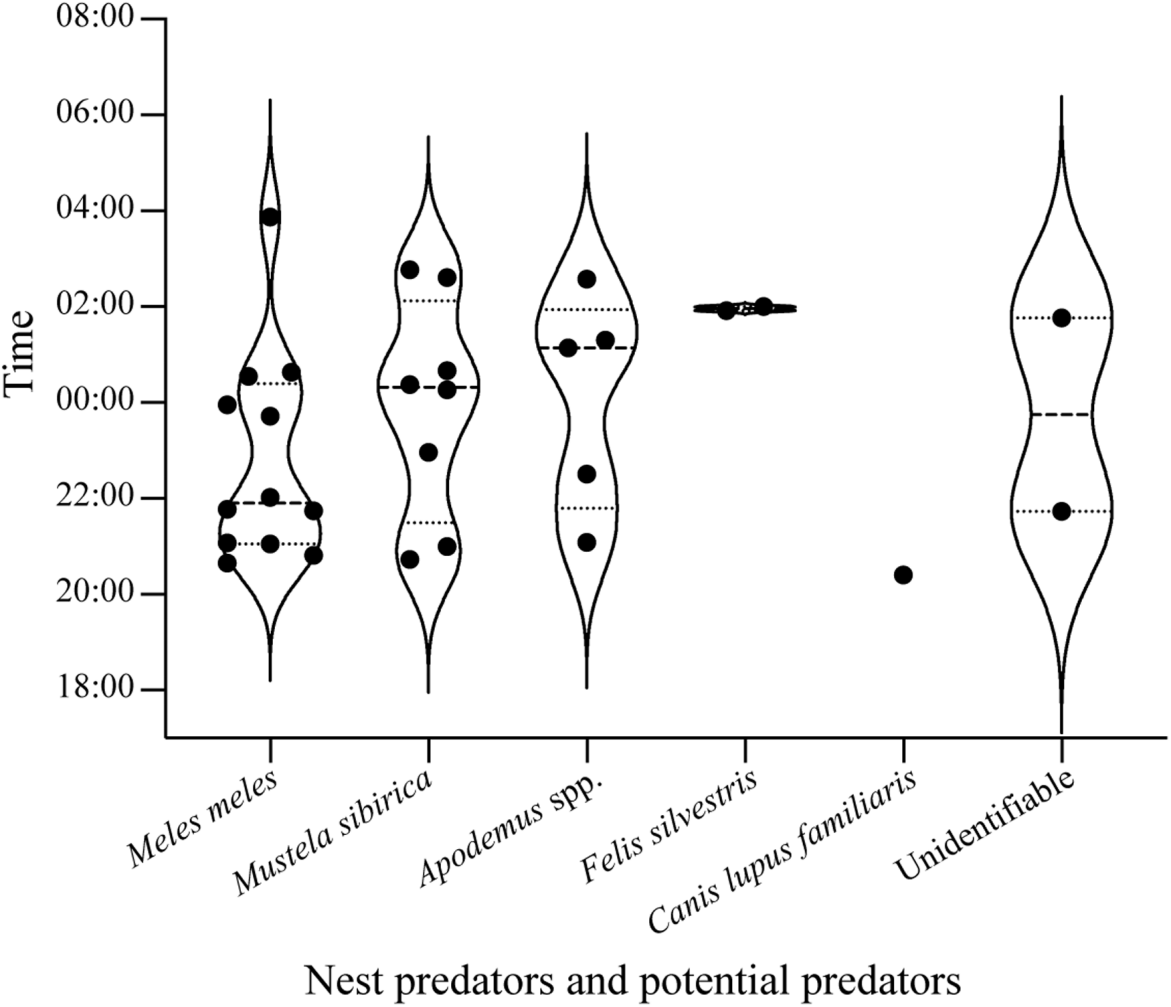
Variations in the predation events by mammal nest predators and potential nest predators that did not initiate the predation recorded by infrared cameras.

**FIGURE 8:**
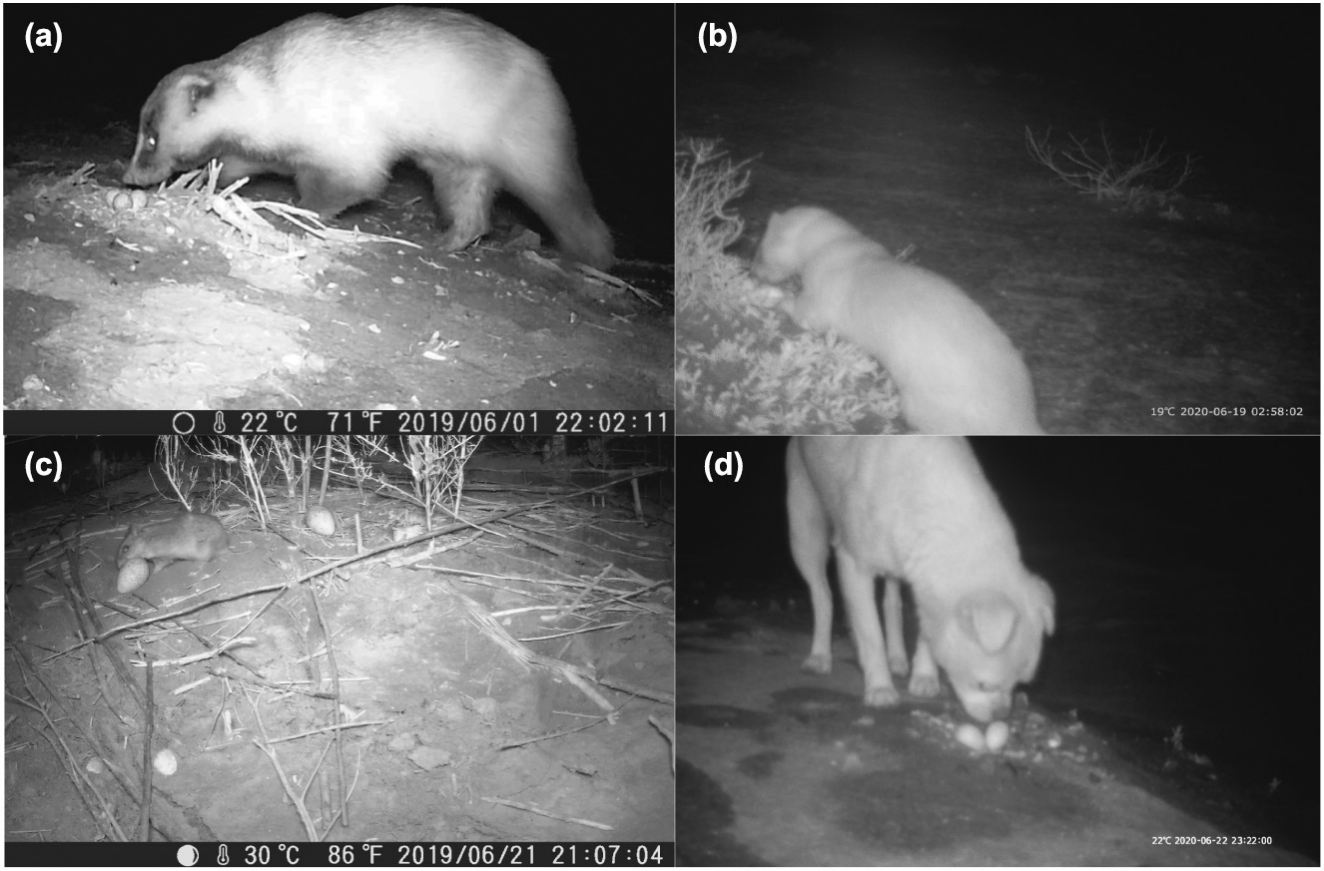
Infrared cameras capturing mammal nest predators in the PA (a: Eurasian badger *Meles meles*; c: rodent *Apodemus* spp.) and non‐PA (b: Siberian weasel *Mustela sibirica*; d: dog *Canis lupus familiaris*).

Infrared‐red cameras recorded higher density or activity of nest predators in the PA (PR = 10.0, CD = 190) than that in the non‐PA (PR = 2.71, CD = 258) (*χ* ^2^ = 9.798, df = 1, *p* = .002). Furthermore, there was a higher nest predation rate in the PA (36.4%, *n* = 33) than in the non‐PA (28.6%, *n* = 21) (Table 5). All confirmed nest predators were mammals, and all predation events occurred from 8:00 p.m. to 04:00 a.m. with a peak at 11:00 p.m. (Figure 7). There was relatively higher species richness and relative abundance of natural mammal nest predators in the PA (Table 5). Three species of nest predators (Eurasian badger *Meles meles*, Siberian weasel *Mustela sibirica* and one species of rodent (*Apodemus* spp.)) were recorded in the PA. In comparison, only one of these wild species (Siberian weasel) was recorded in the non‐PA (Figure 8). Other potential nest predators such as one domestic cat (*Felis silvestris*) and one domestic dog (*Canis lupus familiaris*) were recorded in the PA and non‐PA, respectively, yet no predation events were detected.

**TABLE 5:** The number of predation events (percent of total nests monitored) and potential predators by mammal animals recorded by deployed infrared cameras in a protected area (PA, *n* = 33) at Liaohekou Natural Reserve and adjacent non‐protected area (non‐PA, *n* = 21) around Xiaoling River Estuary, Liaoning, China.

Species	PA		Non‐PA	
	Predation events	Potential predators^a^	Predation events	Potential predators^a^
Siberian Weasel (*Mustela sibirica*)	2 (6.1%)	0	6 (28.6%)	0
Eurasian Badger (*Meles meles*)	7 (21.2%)	5	0	0
Rodent (*Apodemus* spp.)	2 (6.1%)	3	0	0
Cat (*Felis silvestris*)	0	2	0	0
Dog (*Canis lupus familiaris*)	0	0	0	1
Unidentifiable	1 (3.0%)	1	0	0
Total	12 (36.4%)	11	6 (28.6%)	1

^a^ Predator passed the nest but did not predate the nest.

## 4 DISCUSSION

Our study showed no apparent differences in nesting habitats but some variation in nest materials used by breeding Kentish plovers between the PA and non‐PA at an important breeding site in/around the Liaohekou National Nature Reserve. The PA harbored more breeding pairs and had higher nest density than the non‐PA but unexpectedly, the PA breeding group of Kentish plover experienced a much lower nest survival rate than that of the non‐PA birds. Furthermore, there was a relatively lower DSR in *S. salsa* habitat than in the more typical nesting habitat of bare land. However, the effect of habitat switched depending on the nesting substrate, with a lower DSR recorded in bare land nests (*n* = 28) composed of dead *S. salsa* dead stems (*n* = 25). The high encounter rate and species richness of natural nest predators in the PA, such as nocturnal mammals, also likely decreased the nest success rate of these shorebirds.

The establishment of protected areas such as nature reserves or parks is one of the most critical management policies for wildlife conservation (Pringle, 2017; Runge et al., 2015). However, a comprehensive assessment of protected area function and performance is critical to determine the contribution of this practice to preserve unique ecosystems (Ren et al., 2021) or species (such as flagship or endangered species: Sergio et al., 2021). There is growing evidence of a marked increase in population size and survival rates of endangered species in protected areas under strict conservation management policies (Ma et al., 2019; Wang et al., 2014), alongside benefits for other species under the concept of umbrella of flagship species (Rocha et al., 2016; Runge et al., 2019). Yet, at our study site, the protected area did not provide safer breeding habitat for Kentish plovers than the adjacent non‐protected nesting area. In addition, the apparent nest success rate of our Kentish plovers (30.8%) is lower than the nest success rate reported from most other breeding populations (e.g., 45%; Toral & Figuerola, 2012; figure 4 in Que et al., 2015; Tejera et al., 2022). This suggests that the design of the protected area is not benefitting all bird species, including the Kentish plover. However, the PA includes about 80% of the nests from both locations, so although nesting success is relatively higher in non‐protected areas, the contribution of the total number of successful plover nests continues to be much higher within PA. The unexpectedly higher nest survival rate of Kentish plovers in the unprotected nesting area outside the nature reserve also suggests that the importance of this region for breeding birds has been previously overlooked and may represent an important buffer zone for the PA (Lei et al., 2021; Que et al., 2015; Rocha et al., 2016).

The high levels of predation by mammals within the PA, despite optimal nest site selection, may also represent an ecological trap for the plovers (Donovan & Thompson, 2001). This is, in fact, not uncommon in many other protected area ecosystems (Li et al., 2015), and disentangling the drivers of this potential ecological trap, such as the high nest predation rate by mammals within the protected area, would be a crucial first step to improve PA conservation management further. In the recently restored sparse vegetated and bare land that was sampled for this study, we have only found fewer than 10 nests of Saunders's gulls and other tern species within the colony of Kentish plovers, which perhaps implies that there were fewer benefits from any collective defense by these species against mammal predators. Whether they share nest predators between the nest colonies of Saunders's gulls 10 km apart from the Kentish plovers' colonies within the whole protected area merits further investigation. Still, our results should be interpreted cautiously as we assessed plovers' reproductive output, by just focusing on the incubation stage. This is especially relevant in precocial birds, such as our study species, because survival after fledging may influence the reproduction pattern. More research is needed to monitor how the fledgling success of Kentish plovers is affected by the conservation status of their breeding sites.

Ground‐nesting shorebirds, such as plovers and terns, tend to avoid nesting in densely vegetated habitats (Gómez‐Serrano & López‐López, 2014; Norwood, 2011; Swaisgood et al., 2018). Yet, wetland vegetation can be important in certain contexts, especially for populations that experience intense overheating when nesting, where shelter under the vegetation would be necessary for thermoregulation (Lomas et al., 2014; Mayer et al., 2009). Moreover, in addition to providing nesting materials, vegetation can also reduce predation risk through the effect of crypsis (Ekanayake et al., 2015; Engel et al., 2020; Frere et al., 1992). However, our results, showing that nest survival was higher when nests were in bare ground compared to vegetated areas, suggest that adult incubation behaviors may be more influential in this predator community (escape, distraction, or reduced movements to and from nests) than nest concealment from vegetation (Gómez‐Serrano & López‐López, 2014). We acknowledge, however, that other measurable vegetation characteristics (i.e., height and density) are likely to be as important in determining the permeability to the vision of incubating birds.

In this study, we still recorded a substantial proportion of our study population nesting in the *S. salsa* habitat, suggesting birds may also benefit from the reduced risk of eggs overheating within the vegetation (Lomas et al., 2014). Furthermore, previous research on Saunders's gulls, which are mainly dependent on *S. salsa* as nesting habitats in the same area, has suggested that this vegetation is critical in providing shelter for the young gulls shortly after fledging (Tian, 2002). Future research is needed on how *S. salsa* vegetation affects Kentish plovers' offspring survival and parental incubation behavior, thus contributing to the species' population persistence.

The selection of nest materials that enhance nest concealment without impacting thermoregulation is an important selection pressure driving the nest design of ground‐nesting birds (Burhans & Thompson, 2001; Ekanayake et al., 2015; Frere et al., 1992). Nest camouflage relies on matching the visual appearance of the background with nest materials (Gómez et al., 2018; Troscianko, Wilson‐Aggarwal, Spottiswoode, & Stevens, 2016). Nevertheless, studies show that successful and predated nests may not differ in concealment at a microhabitat scale (Bellamy et al., 2018; Koivula & Rönkä, 1998). In this study, nest materials were significantly different between PA and non‐PA plover nests, yet this did not lead to apparent differences in nest survival rates – except for nests made of *S. salsa* stems in non‐vegetated shoreland habitats. We did not quantify the visual matching between the plover's eggs and nest materials. However, from a human vision perspective, it seems reasonable that dry *S. salsa* stems would be more conspicuous in the bare land than other materials (i.e., shell) (Li Donglai, personal observation). Reduced crypticity might significantly contribute to the recorded pattern of lower DSR in the non‐vegetated habitat but not in the *S. salsa* nesting habitat, when nests are predominantly made of dead stems. The relatively higher DSRs in the non‐vegetated shoreland habitat for the nests built with shell and rock nest material also supported the nest crypticity hypothesis, as there was more area of shell bed on the bare land than in the vegetated habitat (Figure 2d). However, we acknowledge that all these inferences related to nest materials need further analysis using avian visual modeling (Gómez et al., 2018; Troscianko, Wilson‐Aggarwal, Stevens, & Spottiswoode, 2016).

Nest predation is a well‐recognized cause of reproductive failure for birds, especially for ground‐nesting birds such as our study species (Ekanayake et al., 2015; Mason et al., 2018), and is strongly related to predator species richness and abundance (Chalfoun et al., 2002). Although we only detected mammalian predators, and despite our small sample size for detecting predation events (*n* = 18), this study revealed higher mammal predator species richness and relative abundance (mostly Eurasian badgers) in the PA compared with the non‐PA. Our results differ from previous studies recording high nest predation pressure from avian (Ekanayake et al., 2015; Engel et al., 2020) or reptilian predators (MacDonald & Bolton, 2008) in other nesting places, but are also consistent with some works in that establishing protected areas may benefit mammal predator communities, leading to the reductions in prey species populations (Naughton‐Treves et al., 2005). The increase in mammalian nest predators within protected areas is thought to occur because of reduced overlap with humans/the potential for human conflict.

We also found that the DSR of Kentish plovers declined throughout the breeding season, especially in PA. Many studies report temporal changes in nest survival rate in the breeding season due to changing temperature, rainfall, social factors, and predation pressure (Hardy & Colwell, 2012; Que et al., 2015; Weintraub et al., 2016). Our findings support the view that more attention should be paid to the effects of predation pressure. More explicitly, Kentish plovers' breeding sites were concentrated in a limited area, which mammal nest predators might quickly locate. Thus, predators could adjust their predation strategies (i.e., developing compelling searching images of nests) and exert high predation pressure on this plover population during the mid and late stages of the breeding season (Gilg et al., 2006; Zhao et al., 2020).

In conclusion, we found a lower daily nest survival rate of Kentish plovers in the PA than in the non‐PA, and relatively high richness and abundance of mammal nest predators. This indicates that PAs may not always function as safer breeding sites for non‐target species. However, if the density of plovers increased considerably during habitat restoration efforts, then the species could do better even with a higher nest predation rate. Moreover, studies on a broader spatial and temporal scale would be needed to confirm these nest site selection patterns that may affect nest success and reproductive effort for species of high conservation value. Furthermore, as for the nest materials, using *S. salsa* dead stems on the bare land contributed to the lower nest survival of Kentish plovers breeding in the Yellow Sea. As a result, the placement of mollusk shells or small gravel for nesting materials should be a useful conservation action aiming to increase the nesting success of Kentish plovers and other shorebirds in the PA. Our research raises the question of to what extent PAs are efficient conservation tools for non‐flagship species that may be affected by unintended changes in animal communities inside these areas, and also highlights that adjacent non‐PAs may also contribute to the conservation of species that are particularly sensitive to predation, which should be addressed in future conservation strategies.

## AUTHOR CONTRIBUTIONS

**Donglai Li:** Conceptualization (equal); data curation (equal); formal analysis (equal); funding acquisition (equal); investigation (equal); methodology (equal); project administration (equal); resources (equal); software (equal); supervision (equal); validation (equal); visualization (equal); writing – review and editing (equal). **Yu Bai:** Data curation (equal); formal analysis (equal); investigation (equal); methodology (equal); writing – original draft (equal). **Weipan Lei:** Conceptualization (equal); methodology (equal); supervision (equal); writing – review and editing (equal). **Pinjia Que:** Conceptualization (equal); investigation (equal); methodology (equal); visualization (equal); writing – review and editing (equal). **Yang Liu:** Conceptualization (equal); data curation (equal); investigation (equal); writing – review and editing (equal). **Emilio Pagani‐Núñez:** Formal analysis (equal); writing – review and editing (equal). **Huw Lloyd:** Writing – review and editing (equal). **Zhengwang Zhang:** Conceptualization (equal); funding acquisition (equal); resources (equal); supervision (equal); writing – review and editing (equal).

## FUNDING INFORMATION

This work was financially supported by Basic Scientific Research Projects of Liaoning Provincial Department of Education (LJKZ0093), the National Natural Science Foundation of China (No. 31911540468 and 31672316 to D.L.), Non‐Profit Foundation of Marine Environment and Ecological Conservation of CNOOC (CF‐MEEC/TR/2021‐14 to Z.Z.), and Shenzhen Mangrove Wetlands Conservation Foundation (MCF: to D.L.).

## CONFLICT OF INTEREST STATEMENT

The authors declare that they have no competing interests.

## Supporting information


Appendix S1

Figure S1

## Data Availability

Study data are publicly available in the Dryad Digital Repository (https://doi.org/10.5061/dryad.6wwpzgn39).
