# Supplementary material for: Mammalian predators and vegetated nesting habitat drive reduced protected area nesting success of Kentish plovers, Yellow Sea region, China
Source: Ecol Evol. 2023 Mar 12;13(3):e9884. doi: 10.1002/ece3.9884 (PMC10008299; doi:10.1002/ece3.9884)
Supplement: Supplementary file 1 — Appendix S1 [file ECE3-13-e9884-s002.docx]

**Supplemental materials**

**Table s1.** The multicollinearity test results between independent variables (model setting 1: including protection status; model setting 2: including distance to the nature reserve) explain the nest DSR of Kentish plover.

| Models | Variables | GVIF | Df |
| --- | --- | --- | --- |
| model setting 1 | Habitat | 2.514494 | 1 |
|  | Nest materials | 1.774623 | 3 |
|  | Nest concealment | 2.650532 | 1 |
|  | Distance to road | 2.324275 | 1 |
|  | Distance to water | 2.164758 | 1 |
|  | Aggregation index | 1.724979 | 1 |
|  | Neighbors distance | 1.284274 | 1 |
|  | Distance to mudflat | 1.326618 | 1 |
|  | Distance to coastline | 2.123146 | 1 |
|  | Protection status | 2.786496 | 1 |
| model setting 2 | Habitat | 2.519133 | 1 |
|  | Nest materials | 1.820518 | 3 |
|  | Nest concealment | 2.652445 | 1 |
|  | Distance to road | 2.299314 | 1 |
|  | Distance to water | 2.178489 | 1 |
|  | Aggregation index | 1.763337 | 1 |
|  | Neighbors distance | 1.28772 | 1 |
|  | Distance to mudflat | 1.299017 | 1 |
|  | Distance to coastline | 2.197135 | 1 |
|  | Distance to PA bournary | 2.953298 | 1 |

**Table s2.** Three alternative models investigating the effects of distance to the boundary of nature reserve, nesting habitats, and nest materials on the daily survival rate of the Kentish plover nests (n=265) during 2018-2021, Liaoning, China. Models are ranked by differences in Akaike's Information Criterion (∆AICc)

| Model | K | logLik | AICc | delta | weight |
| --- | --- | --- | --- | --- | --- |
| S(~Day+Dist_PA+Hab+NM+Hab*NM+AI) | 11 | -334.54 | 691.21 | 0 | 0.46 |
| S(~Day+Dist_PA +Hab+NM+Hab*NM) | 10 | -335.79 | 691.69 | 0.49 | 0.36 |
| S(~Day+Dist_PA+Hab+NM+Hab*NM+AI+Con) | 12 | -334.47 | 693.1 | 1.9 | 0.18 |

**Table s3.** Beta estimates and standard errors with 95% confidence interval (CI) for covariates of daily survival rate of Kentish plover. For abbreviations of covariates can be found in Table 2. The referenced categories for the fixed factors of habitat, and nest material were “bare land” and “plant materials”, respectively.

| **Parameters** | **Estimate** | **SE** | **LCL** | **UCL** | **z value** | ***P*** |
| --- | --- | --- | --- | --- | --- | --- |
| **Intercept** | **2.999** | **0.533** | **1.954** | **4.045** | **5.621** | **< 0.001** |
| **Day** | **-0.024** | **0.007** | **-0.037** | **-0.011** | **3.582** | **< 0.001** |
| **Distance to** PA bournary | **-0.00004** | **0.00001** | **-0.00006** | **-0.00002** | **4.213** | **< 0.001** |
| Hab: vegetation | -0.772 | 0.409 | -1.573 | -0.029 | 1.888 | 0.049 |
| **NM:others** | **1.421** | **0.417** | **0.603** | **2.239** | **3.405** | **0.001** |
| **NM:stones** | **1.958** | **0.470** | **1.037** | **2.879** | **4.166** | **< 0.001** |
| **NM:molluscs shells** | **1.436** | **0.346** | **0.757** | **2.115** | **4.147** | **< 0.001** |
| AI | -0.128 | 0.084 | -0.294 | 0.037 | 1.518 | 0.129 |
| Hab (vegetation):NM(others) | -1.159 | 0.603 | -2.342 | 0.024 | 1.921 | 0.055 |
| **Hab (vegetation):NM(stones)** | **-1.788** | **0.597** | **-2.959** | **-0.617** | **2.993** | **0.003** |
| **Hab (vegetation):NM(molluscs shells)** | **-1.815** | **0.491** | **-2.777** | **-0.852** | **3.694** | **< 0.001** |
| Con | 0.29 | 0.45 | -0.59 | 1.17 | 0.643 | 0.520 |

**
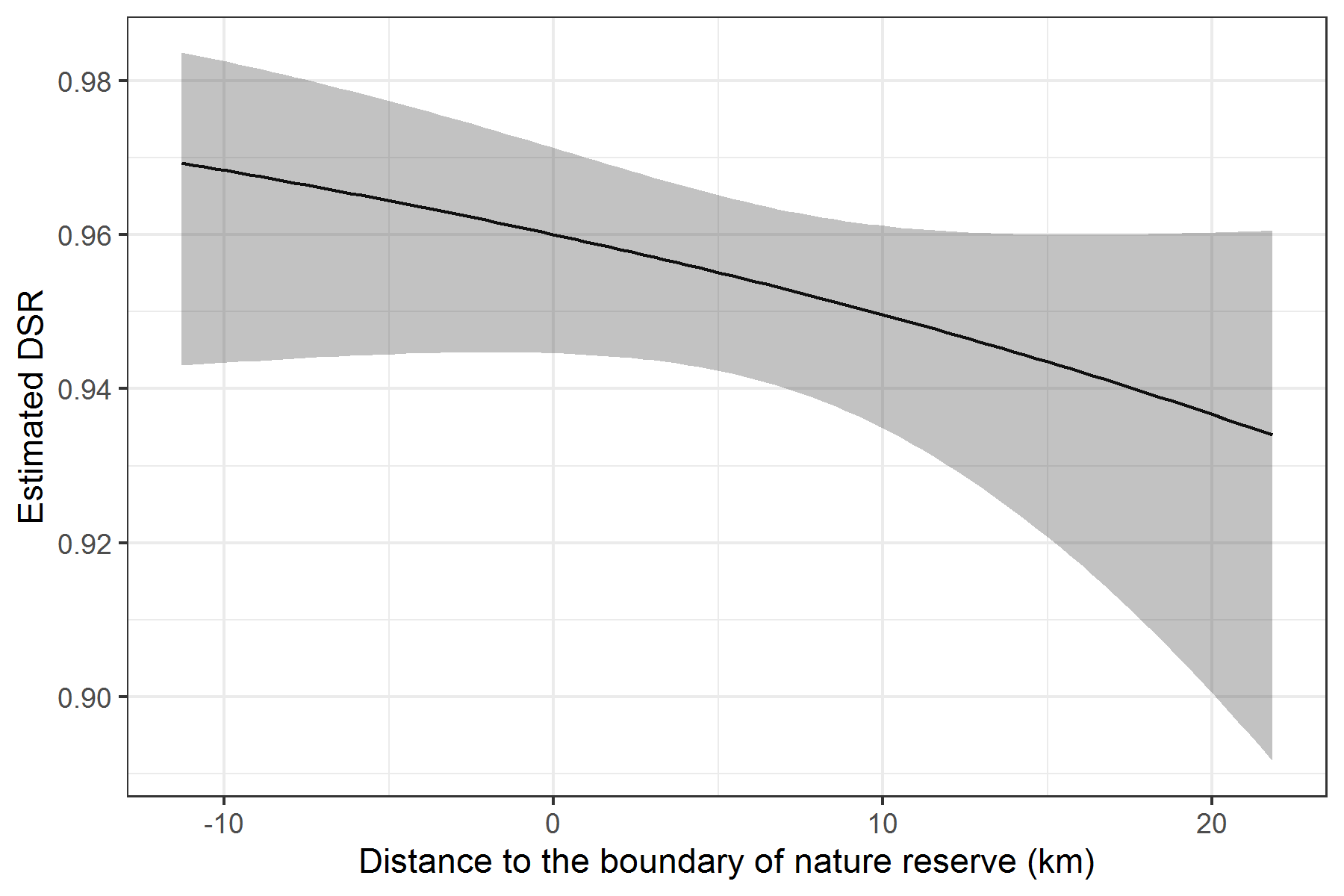
**

**Fig S1.** The estimated DSR of Ketish plovers’ nests declined as the distance to PA boundary increased when inside the nature reserve was a positive value. The gray color represented 95% confidence intervals.
