# Supplementary figures and images for: Mammalian predators and vegetated nesting habitat drive reduced protected area nesting success of Kentish plovers, Yellow Sea region, China
Source: Ecol Evol. 2023 Mar 12;13(3):e9884. doi: 10.1002/ece3.9884 (PMC10008299; doi:10.1002/ece3.9884)

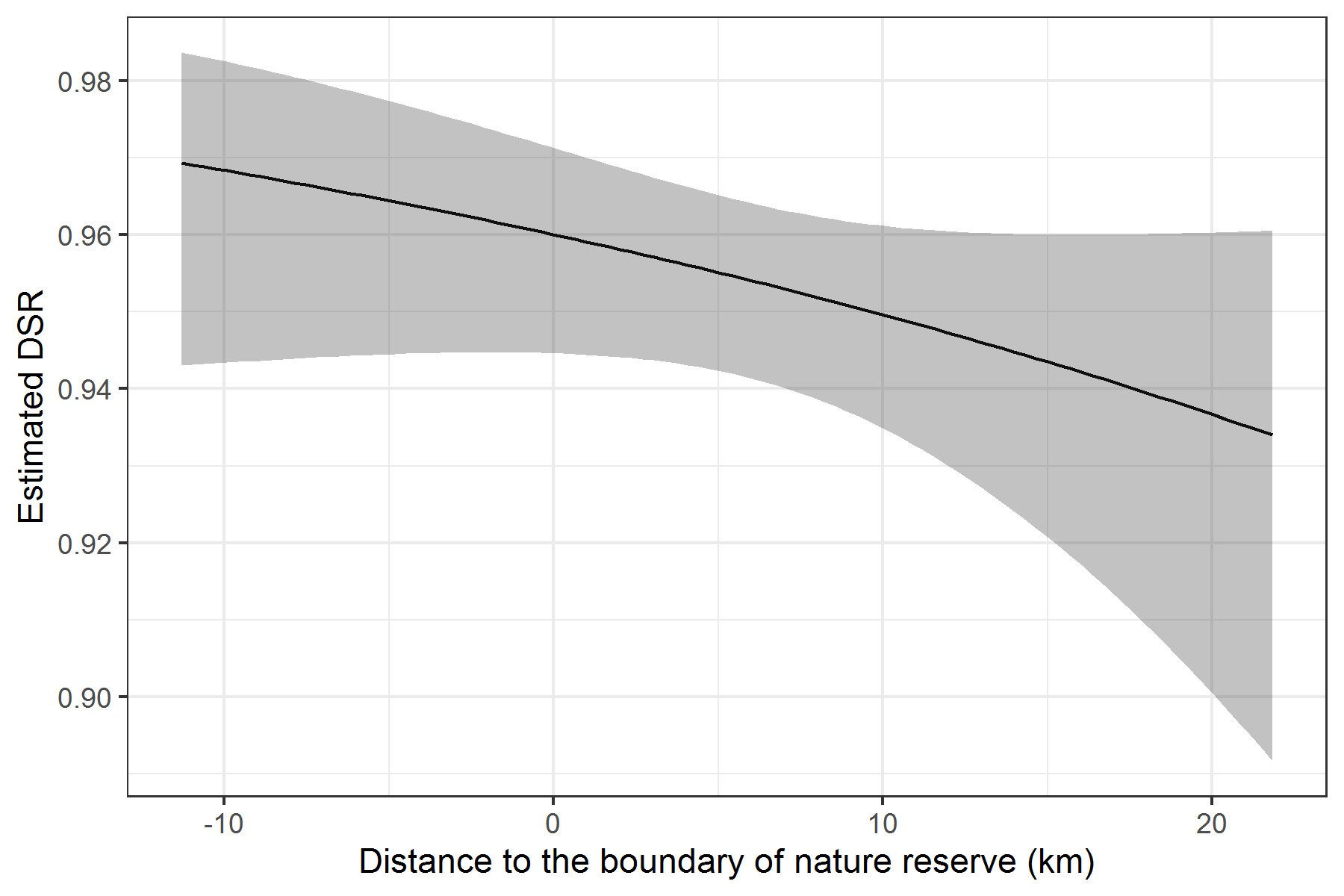

Supplement: Supplementary file 2 — Figure S1 [file ECE3-13-e9884-s001.png]
